# An interpretable machine learning model for predicting distant organ metastasis after radical resection of colorectal cancer

**DOI:** 10.3389/fonc.2026.1764032

**Published:** 2026-02-24

**Authors:** Lin Weibin, Ni Weixiang, Zhou Junwei, Hong Weixuan, Fang Junwei, Wang Meiping, Xiao Chunhong, Huang Guoliang

**Affiliations:** 1 Department of General Surgery, Dongfang Hospital of Xiamen University, School of Medicine, Xiamen University, 900TH Hospital of Joint Logistics Support Force, Fuzhou, China; 2 Department of General Surgery, Fuzong Clinical Medical College of Fujian Medical University, 900TH Hospital of Joint Logistics Support Force, Chinese People’s Liberation Army (PLA), Fuzhou, China; 3 Department of General Surgery, Fuzhou General Teaching Hospital, Fujian University of Traditional Chinese Medicine (900TH Hospital of Joint Logistics Support Force), Fuzhou, China; 4 Department of General Surgery, the 900th Hospital of Joint Logistics Support Force, Fuzhou, China

**Keywords:** colorectal cancer, distant metastasis, explainable machine learning, prediction model, radical resection

## Abstract

**Objective:**

Distant organ metastasis remains the primary factor affecting long-term survival following radical surgery for colorectal cancer (CRC). This study aimed to develop and validate an interpretable machine learning (ML) model to predict the 5-year cumulative risk of distant metastasis after radical CRC surgery.

**Methods:**

A retrospective observational cohort study was conducted using clinical and follow-up data from 341 CRC patients who underwent radical surgery. The cohort was randomly divided into a training set **n*=239* and a validation set **n*=102* at a 7:3 ratio. Feature selection was performed using least absolute shrinkage and selection operator (LASSO) regression, identifying variables associated with the 5-year cumulative occurrence of metastasis. Prediction models were constructed using seven algorithms. Model performance was evaluated through multiple metrics: area under the receiver operating characteristic curve (AUC), accuracy, sensitivity, specificity, F1 score, calibration plots, and decision curve analysis. The SHapley Additive exPlanations (SHAP) method was applied to improve model interpretability.

**Results:**

LASSO combined with tenfold cross-validation selected 11 key features for model development. Among the models tested, the SVM model demonstrated superior performance, achieving a Brier score of 0.144 and an AUC of 0.865 in the validation set. Calibration and clinical decision curves confirmed the SVM model’s strong calibration and clinical applicability. The SHAP dependence plots and force analysis provided explanations at both feature and individual patient levels for the model’s 5-year risk predictions.

**Conclusion:**

This study established a high-accuracy and interpretable ML model capable of effectively predicting the 5-year cumulative risk of distant organ metastasis after radical colorectal cancer surgery, while further external validation is necessary to confirm its clinical utility.

## 1 Introduction

Colorectal cancer (CRC) represents a leading global gastrointestinal malignancy with a continuously rising burden of incidence and mortality (1). The incidence and mortality rates associated with CRC, encompassing both colon and rectal cancers, are remarkably substantial (2, 3). Distant organ metastasis develops in approximately half of patients after CRC resection, invariably leading to a poor prognosis (4). While radical surgery coupled with adjuvant chemotherapy has markedly improved the 5-year survival for early-stage CRC, postoperative distant metastasis persists as the predominant factor driving recurrence and adverse outcomes (5), with the liver and lungs frequently serving as metastatic sites (6). Proactive identification of high-risk individuals and tailored interventions, including intensified monitoring or neoadjuvant treatment strategies, hold the potential to significantly improve survival trajectories. Nevertheless, the current clinical practice is hindered by a lack of accurate risk stratification tools, potentially causing patients to miss critical therapeutic windows.

Emerging evidence underscores the multifactorial etiology of distant metastasis post-CRC resection, implicating factors such as N stage, histological subtype, lymphovascular/perineural invasion, and tumor dimensions (7). TNM-based stratification primarily reflects anatomical staging and provides limited individualized risk assessment for postoperative distant metastasis. Nomograms, derived from Logistic regression or Cox proportional hazards models, have recently demonstrated promise in CRC risk stratification by integrating multivariate predictors for visual assessment. Nevertheless, current models predominantly concentrate on particular metastatic sites (e.g., hepatic metastasis) or radiomic features, thereby overlooking the synergistic utility of pre-operative peripheral blood markers alongside post-operative pathological findings. Limited external validation further curtails their widespread clinical adoption (8). Moreover, a dearth of dedicated models specifically addressing metastasis to “other organs” impedes the advancement of precision medicine objectives.

The Triglyceride-Glucose (TyG) index, a novel biomarker, reflects insulin resistance (IR) (9) and has been implicated in digestive tract malignancies like gastric cancer and pancreatic ductal adenocarcinoma (10, 11). Developed in 2015, the Zhejiang University index (ZJU index) is a non-invasive tool for assessing non-alcoholic fatty liver disease risk and severity, particularly useful for rapid screening in resource-limited settings. While not directly measured in CRC, the ZJU index’s association with obesity and sarcopenia (P<0.05), and its TG component’s role in lipid dysregulation, particularly in obese subgroups, suggest a link to metabolic carcinogenesis pathways (12).The Prognostic Nutritional Index (PNI), a composite measure of systemic inflammation, nutritional status, and immune function, holds significant prognostic value across various cancers (13, 14). The Neutrophil to Albumin Ratio (NPAR), an emerging systemic inflammation marker, integrates indicators of acute inflammation (neutrophil count) and chronic inflammatory states (serum albumin). NPAR is already linked to numerous cardiovascular and metabolic disorders (16), but its specific relevance to colorectal cancer warrants further investigation.The Red Cell Distribution Width to Albumin Ratio (RAR), by combining RDW and serum albumin, reflects multifaceted dysfunction driven by inflammation, oxidative stress, and nutrition. This integrated marker shows potential for predicting mortality and has been associated with adverse outcomes in diverse conditions including acute myocardial infarction, atrial fibrillation, diabetes, heart failure, chronic kidney disease, and stroke (17). The Lymphocyte to HDL Ratio (LHR) has emerged as a potent inflammatory marker, correlated with metabolic syndrome (MetS), cardiovascular risk factors, and sepsis, even outperforming hsCRP and lymphocyte count in MetS diagnosis (18, 19). The Pan-Immune-Inflammation Value (PIV) offers a comprehensive assessment of overall inflammatory burden and immune activity by integrating various immune and inflammatory components (20). The Hemoglobin to Erythrocyte Distribution Width Ratio (HRR), a derived metric from routine blood tests, combines hemoglobin (Hb) with erythrocyte distribution width (RDW) and has demonstrated predictive utility for prognosis in digestive cancers like hepatocellular carcinoma, gastric cancer, and esophageal cancer (21–23). CA19-9, a standard serum biomarker in CRC management, is routinely employed for monitoring recurrence post-surgery and adjuvant chemotherapy, although efforts to enhance its diagnostic specificity and sensitivity remain ongoing (24).

This investigation seeks to develop an interpretable machine learning (ML) framework for predicting the risk of distant organ metastasis following radical colorectal cancer (CRC) resection. Leveraging a comprehensive dataset of patient clinical and pathological information, we have constructed a model designed to discern key factors implicated in metastatic progression. Beyond its predictive accuracy, the model’s inherent interpretability is intended to elucidate the underlying mechanisms driving metastasis, thereby furnishing clinicians with robust, evidence-based insights. Such insights are crucial for tailoring individualized post-operative surveillance and therapeutic interventions, ultimately aiming to enhance patient outcomes.

## 2 Materials and methods

### 2.1 Design and patients

**Figure 1:**
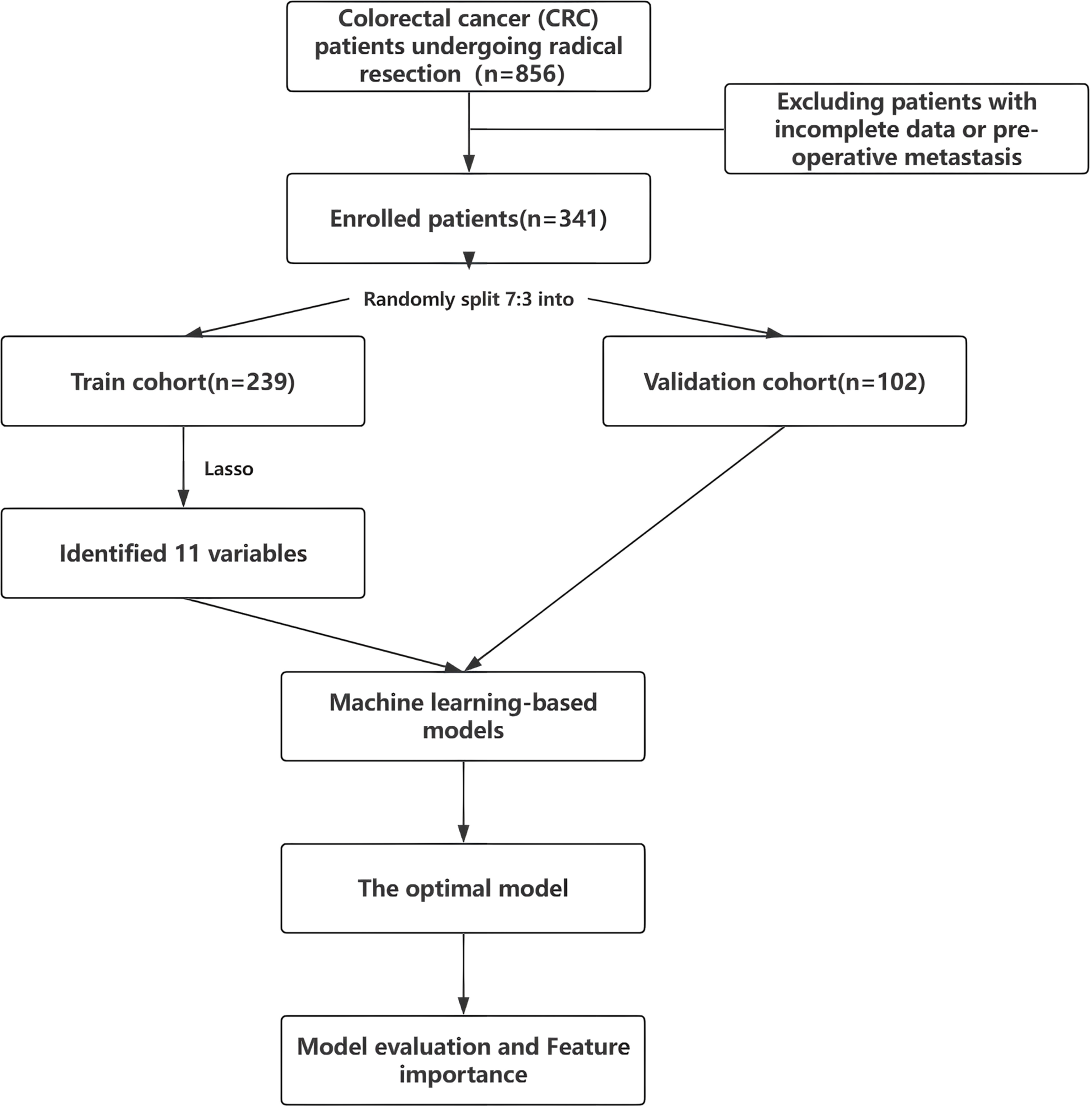
Flowchart outlining patient’s enrollment and study design.

Clinical data from 341 colorectal cancer (CRC) patients undergoing radical resection between December 2015 and December 2022 at the 900th Hospital of the Joint Logistics Support Force of the Chinese People’s Liberation Army were retrospectively collected and followed for up to 5 years postoperatively. Patients were stratified into a metastasis cohort (n=200) and a non-metastasis cohort (n=141) based on the occurrence of distant organ metastasis within the 5-year follow-up period. Inclusion criteria encompassed: (1) pathologically confirmed CRC diagnosis; (2) prior radical surgical intervention for CRC; (3) complete medical records with pre-operative peripheral blood laboratory data and pathological staging; and (4) successful follow-up to ascertain the presence of distant organ metastasis. Exclusion criteria included: (1) evidence of pre-existing distant metastatic disease from pathological or imaging assessments; (2) concurrent other malignancies or significant cardiac, hepatic, pulmonary, or renal dysfunction; (3) hematological disorders; and (4) loss to follow-up. Post-operative distant organ metastasis was diagnosed if any of the following criteria were met: (1) definitive detection of distant CRC metastasis via imaging modalities such as abdominal CT, MRI, or PET-CT; or (2) pathological confirmation of metastatic disease through biopsy or resection of suspicious metastatic lesions. **Figure 1** provides a flowchart illustrating patient recruitment and study design.

### 2.2 Data and variables

The study cohort’s baseline characteristics encompassed demographic data including sex, age, height, and body weight, alongside medical history of diabetes mellitus and hypertension. Tumor-specific parameters were systematically documented, comprising primary tumor site, maximum tumor diameter, TNM stage, histological subtype, and the presence of vascular or nerve invasion. Treatment-related information included the surgical procedure, the number of positive lymph nodes identified, and the administration of adjuvant chemotherapy.”Invasion” was defined as the presence of either perineural invasion or lymphovascular invasion, as confirmed by postoperative pathological examination.

Preoperative laboratory investigations provided critical insights into patients’ physiological status. These included complete blood count parameters such as absolute neutrophil count (ANC), absolute monocyte count (AMC), absolute lymphocyte count (ALC), platelet count (PLT), hemoglobin (Hb), and red blood cell distribution width (RDW). Biochemical markers comprised alanine aminotransferase (ALT), aspartate aminotransferase (AST), triglyceride (TG) level, glucose level (e.g., fasting plasma glucose, FPG), and serum albumin (ALB) level. Additionally, relevant tumor markers, namely carcinoembryonic antigen (CEA) and carbohydrate antigen 19-9 (CA199), were evaluated.

Leveraging these collected peripheral blood parameters, several derived indices were computed to assess inflammatory and nutritional states, potentially correlating with prognosis. These included: the neutrophil-to-lymphocyte ratio (NLR = ANC/ALC), the platelet-to-lymphocyte ratio (PLR = PLT/ALC), and the lymphocyte-to-monocyte ratio (LMR = ALC/AMC). The Zhejiang University Index (ZJU Index) was calculated using the formulas: Male ZJU Index = FPG + BMI + 3 × (ALT/AST) + TG, and Female ZJU Index = FPG + BMI + 3 × (ALT/AST) + 2 + TG. Further indices derived were the red cell distribution width to albumin ratio (RAR = RDW (%)/ALB (g/dL)), the neutrophil percentage to albumin ratio (NPAR = Neutrophil Percentage (%)/ALB (g/dL)), the hemoglobin to red cell distribution width ratio (HRR = Hb (g/L)/RDW (%)), and the lymphocyte to HDL-cholesterol ratio (LHR = ALC (x1000/μL)/HDL-C (mg/dL)). The prognostic index (PIV) was determined as (ANC × AMC × PLT)/ALC, and the triglyceride-glucose index (TyG) was computed as ln[TG (mg/dL) × FPG (mg/dL)/2]. Lastly, the prognostic nutritional index (PNI) was calculated as ALB (g/L) + 5 × ALC, with careful consideration of the specific unit for ALC in the PNI calculation as per standard methodology.

Variables with more than 20% missing data were excluded from the analysis. For variables with less than 20% missing values, missing data were handled using multiple imputation.

### 2.3 Modelling and validation

To develop predictive models for the incidence of distant organ metastasis following curative resection in colorectal cancer patients, logistic regression alongside six machine learning algorithms—namely Decision Tree, Random Forest, XGBoost, LightGBM, Support Vector Machine (SVM), and Artificial Neural Network (ANN)—were employed for model development in the training cohort. Feature selection for variables associated with distant organ metastasis was performed using the Least Absolute Shrinkage and Selection Operator (LASSO) method coupled with tenfold cross-validation. The identified significant variables subsequently served as inputs for training and optimizing the models. Model parameters were optimized through random hyperparameter search following 5-fold cross-validation and 100 bootstrap iterations, with the objective of maximizing the area under the receiver operating characteristic curve (AUC). The predictive performance of the developed models was rigorously evaluated in an independent validation cohort using the Brier score, AUC, calibration curves, and decision curves. The Brier score, ranging from 0.00 to 1.00, indicates better calibration as it approaches 0.00.

### 2.4 Model interpretability

SHAP (SHapley Additive exPlanations) is a robust methodology for interpreting the predictions of machine learning (ML) models. Rooted in cooperative game theory, specifically Shapley values, SHAP attributes a unique contribution to each feature for a given model prediction. The magnitude of a SHAP value quantifies a feature’s impact; larger absolute SHAP values indicate a greater influence on the model’s output, thus signifying higher feature importance. Conversely, smaller absolute SHAP values suggest a lesser impact. Furthermore, the sign of a SHAP value provides directional information: positive SHAP values denote a favorable impact, driving the prediction higher, whereas negative SHAP values represent an adverse effect, leading to a reduced prediction value.

## 3 Results

### 3.1 Characteristics of the study population

The study encompassed 341 participants. Baseline characteristics, including clinical-pathological features and laboratory findings, were compared between the training and validation cohorts. While a significant difference was detected in the administration of neoadjuvant chemotherapy (P = 0.02), all other assessed variables exhibited no statistically significant variation between the two groups (P>0.05), as detailed in **Table 1**.

**Table 1:** Characteristics of the training and validation sets.

Characteristics	Training set (n=239)		Validation set (n=102)	P -value
T, n (%)^a^				0.129
T_1_		21 (8.79)	8 (7.84)	
T_2_		55 (23.01)	20 (19.61)	
T_3_		117 (48.95)	45 (44.12)	
T_4_		46 (19.25)	29 (28.43)	
N, n (%)^b^				0.733
N_0_		103 (43.10)	46 (45.10)	
N_1_~N_2_		136 (56.90)	56 (54.90)	
Gender, n (%)^b^				0.842
Male		127 (53.14)	53 (51.96)	
Female		112 (46.86)	49 (48.04)	
Hypertension, n (%)^b^				0.101
No		208 (87.03)	95 (93.14)	
Yes		31 (12.97)	7 (6.86)	
Diabetes, n (%)^c^				0.314
No		227 (94.98)	100 (98.04)	
Yes		12 (5.02)	2 (1.96)	
Tumor Location, n (%)^a^				0.584
Right hemicolon		53 (22.18)	29 (28.43)	
Left hemicolon		73 (30.54)	25 (24.51)	
Rectum		113 (47.28)	48 (47.06)	
Pathology, n (%)^b^				0.881
Common		145 (60.67)	61 (59.80)	
Other		94 (39.33)	41 (40.20)	
Invasion, n (%)^b^				0.784
No		156 (65.27)	65 (63.73)	
Yes		83 (34.73)	37 (36.27)	
Surgical Approach, n (%)^b^				0.623
Open		87 (36.40)	40 (39.22)	
Laparoscopy		152 (63.60)	62 (60.78)	
Postoperative Chemotherapy, n (%)^b^				0.020
No		60 (25.10)	14 (13.73)	
Yes		179 (74.90)	88 (86.27)	
CEA, n (%)^b^				0.671
Negative		137 (57.32)	61 (59.80)	
Positive		102 (42.68)	41 (40.20)	
CA199, n (%)^b^				0.173
Negative		143 (59.83)	69 (67.65)	
Positive		96 (40.17)	33 (32.35)	
Lymph nodes retrieved^a^		18.00 (13.00, 25.00)	19.50 (14.00, 29.75)	0.133
Tumor Maximum^a^		4.50 (3.50, 5.50)	4.50 (3.50, 5.50)	0.574
Age^d^		43.41±5.58	42.75±5.96	0.328
Height^d^		1.64±0.07	1.65±0.07	0.453
Weight^d^		60.18±10.38	60.56±12.43	0.768
BMI^d^		22.21±3.07	22.02±3.25	0.611
NEU^a^		4.04 (3.02, 5.50)	3.59 (2.65, 5.29)	0.160
MONO^a^		0.39 (0.31, 0.51)	0.40 (0.27, 0.51)	0.355
LYM^a^		1.63 (1.30, 2.05)	1.67 (1.40, 2.14)	0.586
Plt^a^		266.00 (214.00, 335.00)	263.50 (205.50, 344.50)	0.654
Hb^d^		119.99±25.37	117.81±26.97	0.477
RDW^d^		14.32±2.19	14.27±2.24	0.848
AST/ALT^a^		1.60 (1.10, 2.37)	1.46 (1.10, 2.14)	0.603
TG^a^		1.03 (0.81, 1.46)	1.06 (0.82, 1.27)	0.535
HDL^d^		1.15±0.32	1.15±0.30	0.844
Glu^d^		5.21±1.19	5.10±1.11	0.430
Alb^d^		41.90±4.57	41.81±4.40	0.868
ZJU^a^		27.57 (15.33, 31.68)	26.60 (14.16, 31.96)	0.727
NLR^a^		2.50 (1.71, 3.33)	2.20 (1.60, 3.18)	0.132
PLR^a^		167.50 (122.31, 217.27)	156.30 (120.78, 222.68)	0.395
LMR^a^		4.30 (2.88, 5.65)	4.58 (3.15, 6.08)	0.200
HRR^d^		8.68±2.61	8.61±2.72	0.818
TyG/Glu^a^		0.21 (0.17, 0.28)	0.21 (0.17, 0.27)	0.867
PNI^d^		52.54±7.61	52.27±7.59	0.761
NPAR^a^		16.00 (12.00, 20.00)	16.00 (12.00, 21.75)	0.979
RAR^d^		0.35±0.07	0.35±0.08	0.995
LHR^a^		1.80 (1.26, 2.58)	2.08 (1.33, 2.72)	0.237
PIV^a^		245.34 (144.61, 449.57)	227.93 (105.66, 423.95)	0.117

^a^Mann-Whitney-Wilcoxon test; ^b^Pearson's chi-squared test; ^c^Chi-squared test with continuity correction; ^d^Independent samples t-test.

### 3.2 Development of models

Eleven clinical features, namely N, Pathology, Invasion, Surgical Approach, CEA, CA199, Tumor Maximum, ZJU, PNI, NPAR, and LHR, were identified as significantly correlated with distant organ metastasis post-radical resection in colorectal cancer patients, employing the least absolute shrinkage and selection operator (LASSO) coupled with tenfold cross-validation.Based on LASSO regression, these eleven features were retained as predictive variables associated with the outcome.These eleven features served as predictors for developing models using logistic regression and six machine learning algorithms. Optimal hyperparameters for each trained and optimized model were determined (**Table 2**). Although powerful, Random Forest, XGBoost, and LightGBM were excluded due to their propensity for overfitting, which might impair their predictive capability on novel datasets. Our overarching goal was to construct a model that achieved a judicious balance of predictive accuracy, interpretability, and generalization robustness. On the training data, LR [AUC = 0.883 (95% CI 0.839–0.921)], DT [AUC = 0.888 (95% CI 0.848–0.924)], ANN [AUC = 0.851 (95% CI 0.799–0.892)], and SVM [AUC = 0.878 (95% CI 0.832–0.917)] exhibited robust predictive power (all P < 0.001). Correspondingly, calibration curves from the training set indicated Brier scores of 0.154 for LR, 0.192 for DT, 0.183 for XGBoost, 0.144 for SVM, and 0.177 for ANN.

**Table 2:** Full super-parameters of techniques.

Model and Optimal Parameters
DT: 'ccp_alpha': 0.0, 'max_depth': 10, 'max_features': 'sqrt', 'min_samples_split': 20 RF: n_estimators = 100 , max_features = 2 XGBoost: {'learning_rate': 0.2, 'max_depth': 3, 'n_estimators': 50, 'subsample': 0.6} LightGBM: {'colsample_bytree': 0.8, 'learning_rate': 0.2, 'n_estimators': 100, 'num_leaves': 31, 'subsample': 0.6} SVM:{'C': 0.1, 'degree': 2, 'gamma': 'scale', 'kernel': 'linear'} ANN: {'activation': 'logistic', 'hidden_layer_sizes': (100,)}

### 3.3 Model validation

**Figure 2:**
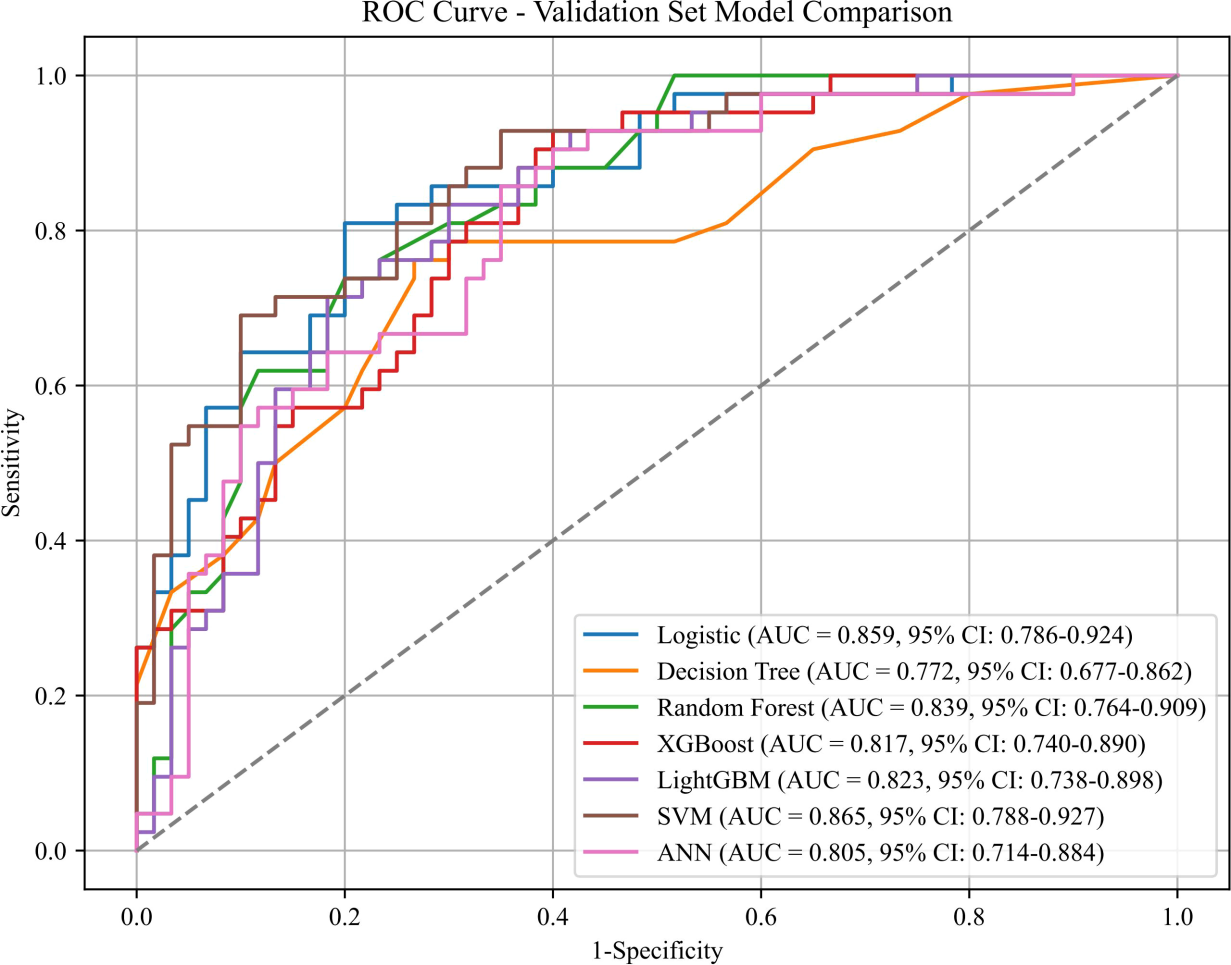
The receiver operating characteristics curves of the validation set.

**Figure 3:**
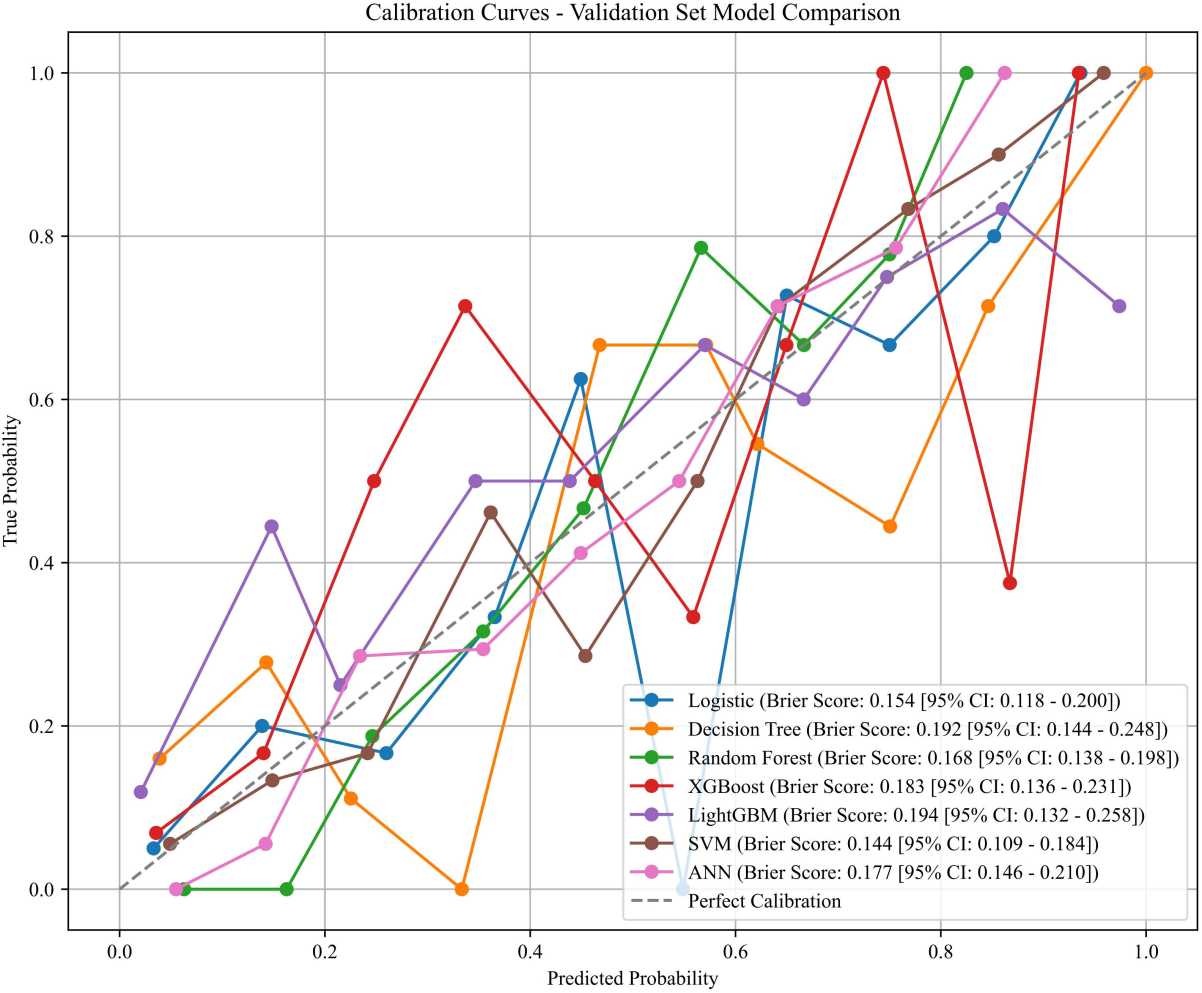
The calibration curves of the training set.

Within the validation set, the SVM model emerged as the leading performer based on aggregate metrics, exhibiting an AUC of 0.865 (95% CI 0.788–0.927) (**Figure 2**), a Brier score of 0.144 [95% CI: 0.109 - 0.184] (**Figure 3**). These performance metrics reflect internal validation results derived from the held-out validation cohort. Although the SVM model demonstrated an acceptable overall calibration as indicated by the Brier score, the calibration curve exhibited local variability across certain probability ranges, particularly in the mid-range. This deviation from the ideal line may be partly attributed to the limited number of samples within specific probability bins, which can lead to unstable empirical estimates. In addition, the inherent margin-based nature of SVMs may contribute to less reliable probability estimates at intermediate thresholds. Nevertheless, the calibration curve generally followed the diagonal trend, especially at higher predicted probabilities, suggesting that the model remains reasonably calibrated for clinically relevant risk stratification within this single-center retrospective cohort. F1-score of 0.734 (**Table 3**).

**Table 3:** Discrimination indicators of the predictive models in the training and validation sets.

Model	AUC ( 95% CI)	Accuracy	Precision	Sensitivity	Specificity	F1 score	Youden's J	PPV	NPV
Logistic	0.859(0.786~0.924)	0.765	0.750	0.643	0.850	0.692	0.493	0.750	0.773
Decision Tree	0.772(0.677~0.862)	0.716	0.667	0.619	0.783	0.642	0.402	0.667	0.746
Random Forest	0.839(0.764~0.909)	0.765	0.765	0.619	0.867	0.684	0.486	0.765	0.765
XGBoost	0.817(0.740~0.890)	0.706	0.650	0.619	0.767	0.634	0.386	0.650	0.742
LightGBM	0.823(0.738~0.898)	0.755	0.718	0.667	0.817	0.691	0.483	0.718	0.778
SVM	0.865 (0.788~0.927)	0.794	0.784	0.690	0.867	0.734	0.557	0.784	0.800
ANN	0.805(0.714~0.884)	0.745	0.711	0.643	0.817	0.675	0.460	0.711	0.766

**Figure 4:**
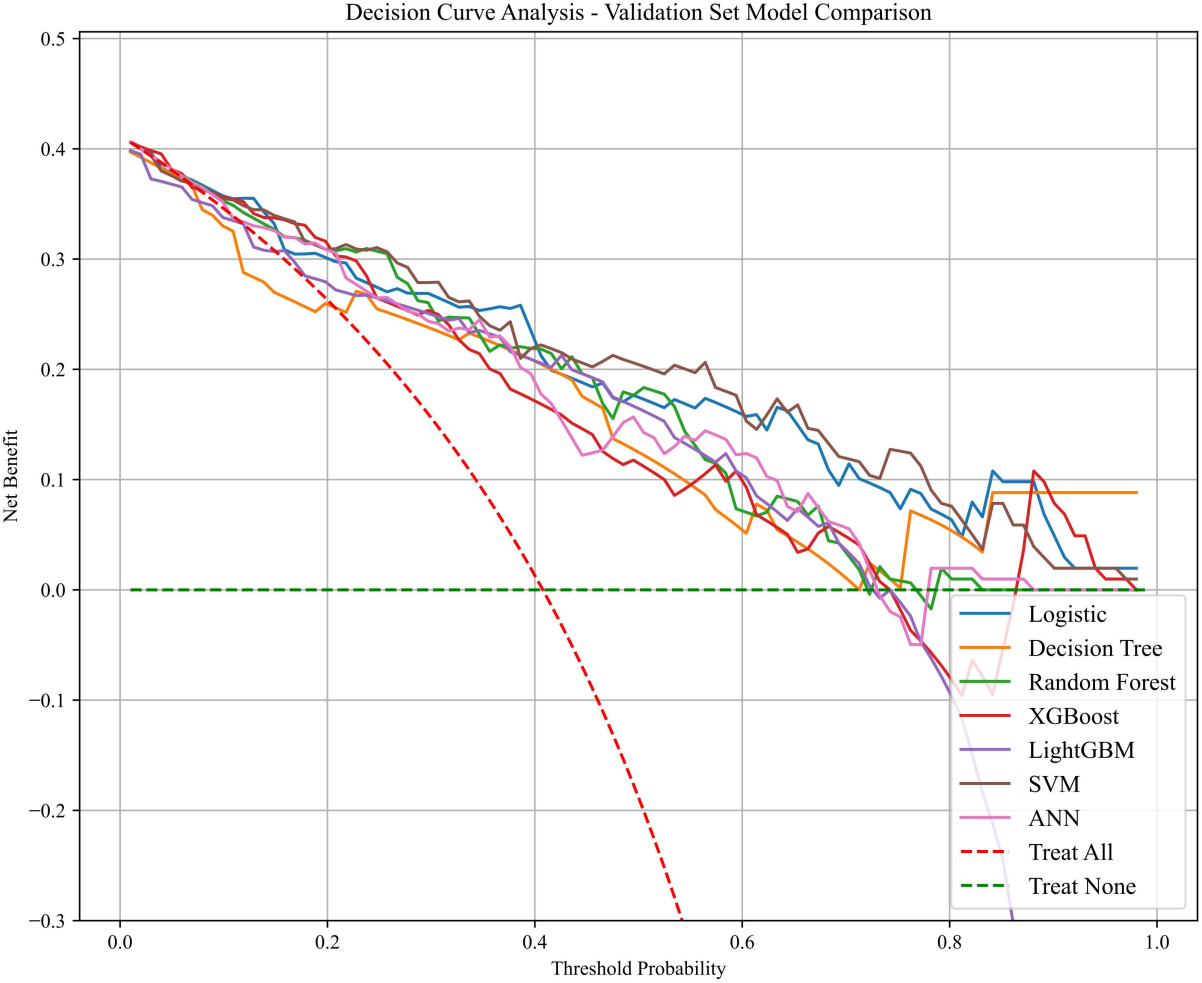
Clinical decision curve analysis of each model.

Clinical decision curve analysis reinforced the SVM model’s practical clinical applicability. Based on the decision curve analysis, the model demonstrates a superior net benefit compared with the ‘treat-all’ and ‘treat-none’ strategies across a threshold probability range of 0.10–0.70, with the greatest clinical value observed between 0.15 and 0.45. This range covers clinical decision-making needs from low- to intermediate-risk patients, suggesting that the model may help avoid overtreatment in low-risk patients while ensuring timely intervention for high-risk individuals under internal validation conditions (**Figure 4**).

**Figure 5:**
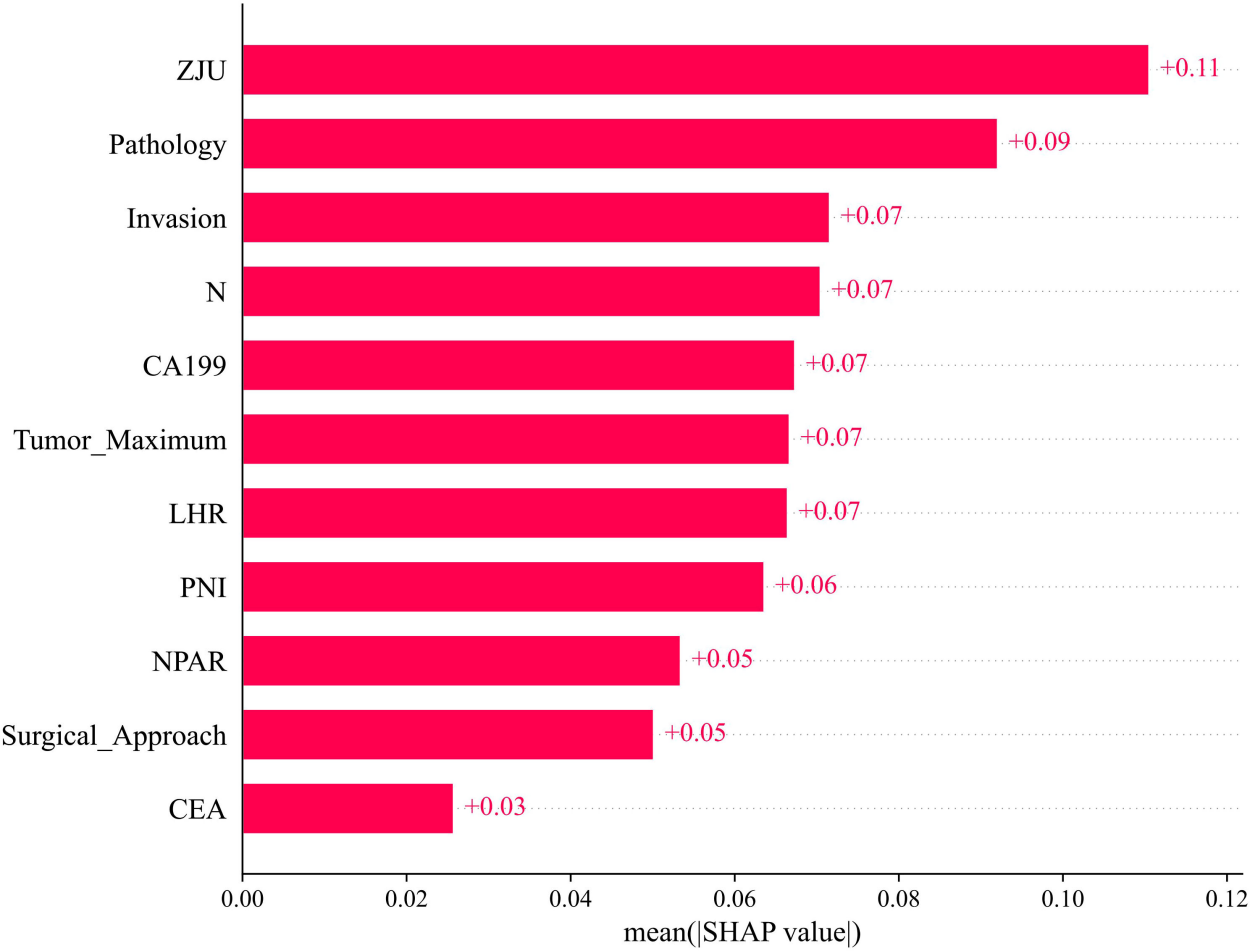
Analysis of feature importance based on SHAP summary plot.

**Figure 6:**
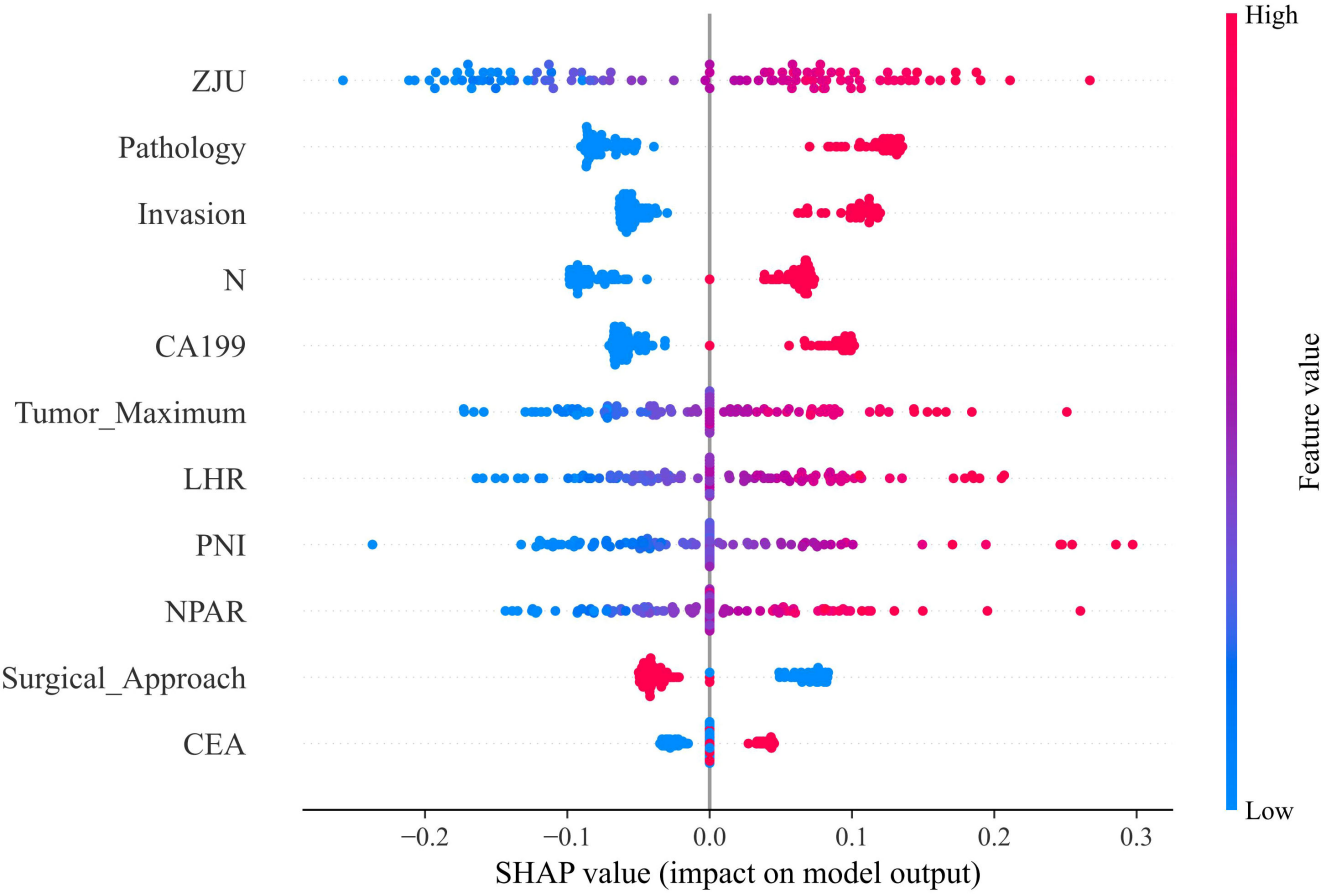
The importance of each feature based on the SHAP values of the training set. Each line in the y -axis represents a feature and the x -axis represents the SHAP value of the feature. Each dot represents a participant and the feature value is indicated by the colour, with red indicating a higher value and blue indicating a lower value. Positive feature-SHAP correlation indicates a risk factor for post-operative distant organ metastasis, while negative correlation indicates a protective factor.

Building upon the SVM framework, we investigated the importance of the identified clinical features in predicting distant organ metastasis for the entire patient population. Analysis of both individual and averaged SHAP values identified ZJU, Pathology, and Invasion as the three features contributing most prominently to the model’s predicted risk of post-radical surgery distant metastasis in colorectal cancer patients (**Figure 5** & **6**).

**Figure 7:**
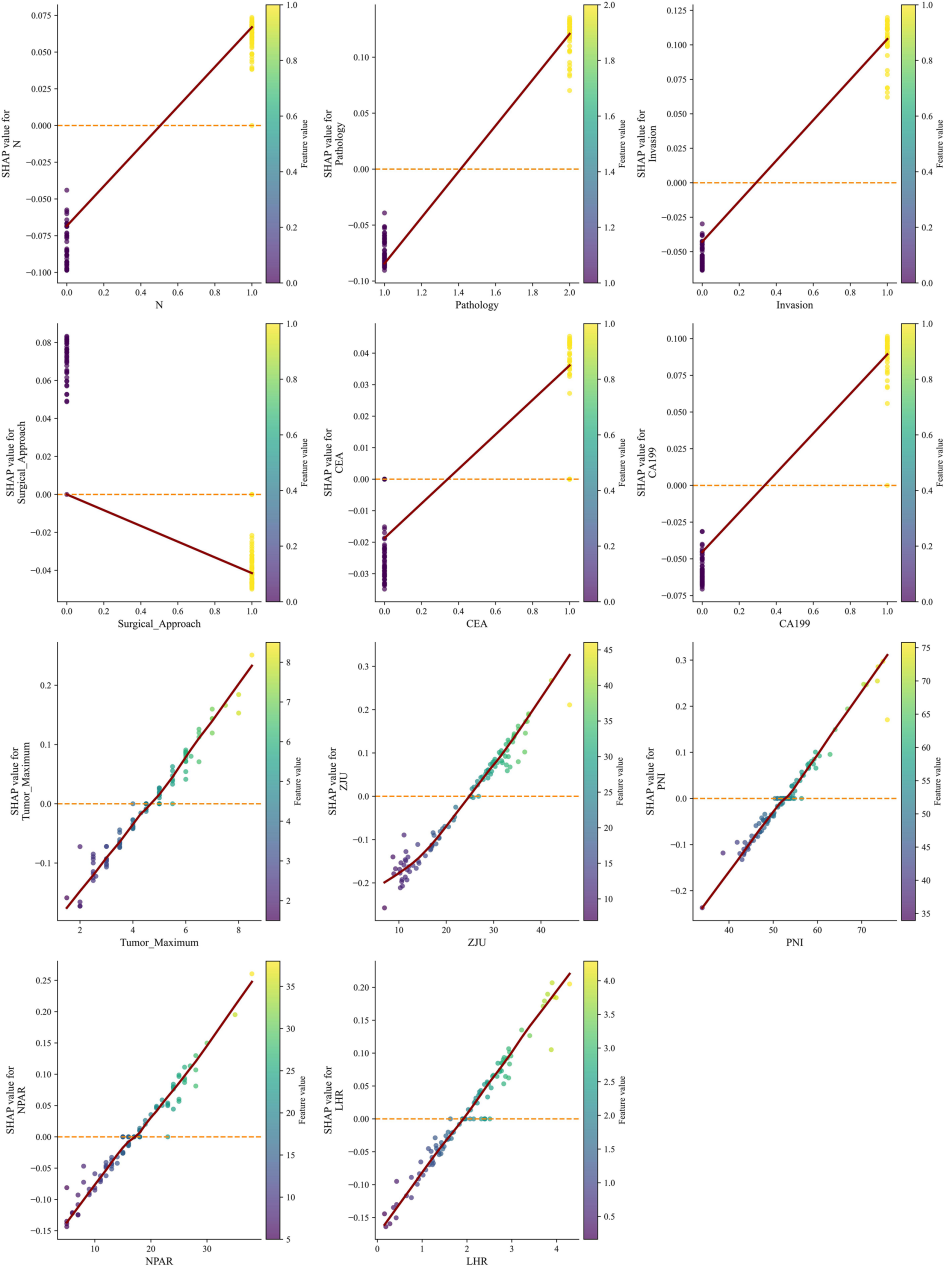
This figure demonstrates the feature dependence of six critical features within the model: N, Pathology, Invasion, Surgical_Approach, CEA, and CA199.

To gain deeper insights into the interplay between feature values and their predictive contributions, and to identify potential feature interactions, partial dependence plots were constructed. These plots effectively depict the impact of a feature’s specific value range on the model’s predicted output (as reflected by Shapley values), and whether this impact is conditional on the value of another feature (**Figure 7**).

## 4 Discussion

Inflammation is closely implicated in colorectal cancer (CRC); inflammatory cells, via cytokine secretion, alter the tumor microenvironment (TME) to facilitate tumor initiation, growth, advancement, and dissemination (25). CRC patients face a high risk of postoperative recurrence and distant spread. Traditional TNM staging and tumor invasion metrics offer an incomplete picture of disease status. Emerging research consistently highlights the inseparable relationship between the inflammatory and nutritional states of CRC patients and their disease trajectory, with nutritional status serving as an indicator of immune competence. The tumor-promoting inflammatory mechanisms share similarities with physiological inflammatory responses during development, immunity, tissue homeostasis, or repair (26). Therefore, this study seeks to explore the predictive value of various novel composite biomarkers reflecting inflammation and metabolic status for predicting distant organ metastasis after CRC surgery.

This study successfully developed and validated a machine learning model based on Support Vector Machine (SVM) that exhibits both high predictive accuracy and interpretability for assessing the risk of distant organ metastasis following radical resection in colorectal cancer (CRC) patients. Through retrospective analysis of data from 341 patients, the LASSO regression method was employed to identify 11 key clinical features, including N stage, Pathology, and Invasion, which were subsequently used to construct various predictive models. Comparative performance evaluation revealed that the SVM model demonstrated the most outstanding predictive capability in the validation set, with its AUC (0.865) and Brier score (0.144) outperforming other models. Furthermore, rigorous assessment via calibration curves and clinical decision curve analysis confirmed its favorable calibration and clinical utility. The SHAP analysis further indicated that features such as ZJU, pathological type, and the degree of tumor invasion had high contributions to the model’s prediction of distant organ metastasis risk, providing intuitive explanations of the model predictions at both the feature and individual levels.

Independent risk factors identified in this study included N stage, pathological features, tumor invasion, surgical approach, CEA, CA199, maximal tumor size, ZJU, PNI, NPAR, and LHR. These factors significantly contribute to the development of distant metastases post-colorectal cancer resection. Specifically, N staging quantifies lymph node involvement, a primary pathway for distant spread. Perineural invasion underscores tumor aggressiveness, thereby increasing the probability of metastasis. Moreover, elevated serum CEA and CA199 levels, known indicators for colorectal cancer, correlate closely with tumor invasiveness and metastatic risk.

A significant contribution of this study is the introduction of novel composite indices, including ZJU, NPAR, and LHR. These metrics were constructed by combining peripheral blood parameters and pathological characteristics to achieve a more holistic assessment of patients’ inflammatory state, metabolic derangements, and immune status. For example, The ZJU index is a recently proposed composite metabolic parameter that integrates multiple indicators, including triglycerides (TG), aspartate aminotransferase (AST), alanine aminotransferase (ALT), body mass index (BMI), and fasting blood glucose (FBG). By combining these variables, the ZJU index provides a more comprehensive assessment of metabolic health than isolated biochemical markers and enables more effective identification of metabolic dysfunction and its associated health risks. Previous studies have demonstrated that the ZJU index is closely associated with lipid dysregulation, insulin resistance, and obesity, and has also been linked to an increased risk of metabolic -related conditions such as non -alcoholic fatty liver disease (NAFLD) and obstructive sleep apnea (OSA) (12). NPAR, reflecting systemic inflammation, integrates neutrophil count with albumin levels.The NPAR, as a novel indicator of inflammatory and nutritional status, has attracted increasing attention in recent clinical research. Its applications span a wide range of conditions, including disease diagnosis, prognostic assessment, and prediction of therapeutic response. By integrating the neutrophil percentage, which reflects systemic inflammatory activity, with serum albumin levels, which indicate nutritional status and are also influenced by systemic inflammation, NPAR is considered to provide a more comprehensive representation of a patient’s overall pathophysiological condition (15, 16). Likewise, The LHR has been identified as a novel inflammatory marker and has been shown to be associated with metabolic syndrome (MetS), a prothrombotic and pro -inflammatory condition, as well as cardiovascular risk factors and sepsis. Moreover, LHR has demonstrated superior performance compared with high -sensitivity C -reactive protein (hsCRP) and lymphocyte count in identifying the presence of MetS (18, 19). Although the ZJU index, NPAR, and LHR have limited validation in colorectal cancer (CRC)–specific studies, there is a plausible biological rationale for their inclusion. The ZJU index reflects metabolic and hepatic dysregulation associated with chronic inflammation and insulin resistance, while NPAR and LHR integrate systemic inflammation, immune status, lipid metabolism, and nutritional condition, all of which are closely involved in CRC development and progression. Collectively, these composite indices capture complementary aspects of metabolic and inflammatory alterations relevant to tumor biology. Nevertheless, further prospective studies are required to validate their clinical utility in CRC populations.

In this study, we developed a highly accurate and interpretable SVM-based machine learning model for predicting distant organ metastasis following curative colorectal cancer (CRC) resection. Critical risk predictors were identified through LASSO feature selection and their contributions to model predictions were further characterized using SHAP analysis, facilitating personalized risk assessment. The model demonstrates strong predictive power and clinical utility, providing a valuable tool for the individualized evaluation and precise management of post-operative CRC metastatic risk.

Several recent studies have applied machine−learning or deep−learning approaches to predict metastasis in colorectal cancer (CRC), providing an important context for our work. Kang et al. developed a LASSO−based prediction model incorporating tumor−infiltrating lymphocytes (TILs) and histopathologic features to estimate lymph node metastasis risk in patients with T1 CRC, demonstrating improved performance over conventional Japanese criteria. Their study highlights the value of immune−related and pathologic variables combined with interpretable machine−learning methods in a well−defined early−stage CRC population (27). In contrast, Li et al. proposed an interpretable deep−learning fusion framework integrating natural language processing of free−text electronic health records with structured laboratory data to predict postoperative liver metastasis, achieving strong predictive performance but requiring large−scale datasets, complex model architectures, and advanced data preprocessing (28). Compared with these deep−learning–based approaches, our SVM−based model emphasizes transparency, robustness, and ease of implementation by relying on routinely available clinical and laboratory variables, thereby offering a more pragmatic balance between interpretability and clinical utility, particularly in settings where multimodal data or large annotated datasets are not readily accessible. Together, these studies suggest that different modeling strategies may be complementary, with model choice best guided by clinical context, data availability, and intended application.

## 5 Limitations

This study, while yielding important insights, has inherent limitations. Firstly, its retrospective, single-center design based on 341 cases limits broad applicability. Although LASSO was employed to minimize overfitting during feature selection, external validation in larger, multi-center prospective cohorts is crucial to confirm the model’s generalizability. With a limited sample size, reliance on a single train–test split may indeed lead to overestimation of model performance; alternative approaches such as repeated cross-validation or a fully bootstrap-based modeling strategy could further enhance the robustness of internal validation.Secondly, the current feature set may not fully capture all determinants of distant metastasis, including the tumor microenvironment, genomic alterations, and patient lifestyle factors. Incorporating these missing elements could potentially refine predictive performance. Additionally,A major limitation of this study is the lack of precise follow-up time data; consequently, we employed a binary logistic regression model instead of survival analysis models (such as the Cox proportional hazards model) to predict distant metastasis. This approach ignores the time factor, which may lead to information loss and an inability to handle censored data. However, our model aims to utilize clinical pathological features available immediately after surgery to predict the eventual risk of distant metastasis, which still holds significant guiding value for the selection of adjuvant therapy. Future studies will incorporate prospective follow-up time data to establish time-to-event prediction models.Lastly, the follow-up period may impact the assessment of long-term metastatic risk.

Future research should expand the sample size and incorporate multi-center data to enhance the model’s applicability. Furthermore, integrating molecular biology and radiomics techniques to explore additional potential risk factors, such as gene mutations, microRNA expression, and tumor microenvironment characteristics, may improve the model’s predictive capability. Concurrently, conducting external validation studies to assess the model’s performance across diverse populations and healthcare settings will provide crucial evidence for clinical implementation.

## 6 Clinical implications

Clinically, this model may serve as a postoperative risk stratification tool for patients with colorectal cancer after curative resection. Patients predicted to be at high risk of distant metastasis could be considered for intensified surveillance, including closer imaging follow-up and tumor marker monitoring, or earlier evaluation for adjuvant systemic therapy. In contrast, low-risk patients may benefit from reduced follow-up intensity, potentially avoiding unnecessary examinations and overtreatment.

The model is intended to support, rather than replace, clinical decision-making. Misclassification may lead to potential risks, such as increased patient anxiety and overtreatment in false-positive cases, or delayed detection of metastasis in false-negative cases. Therefore, the model should be applied in conjunction with established clinical judgment.The SHAP-based interpretability illustrates how individual features contribute to model predictions rather than establishing causal relationships, but prospective multicenter validation is required before routine clinical implementation.

## 7 Conclusions

This study successfully developed a highly accurate and interpretable Support Vector Machine (SVM) model for predicting the 5-year cumulative risk of distant organ metastasis following curative resection of colorectal cancer (CRC). Through the application of LASSO for feature selection and SHAP for model interpretation, key risk predictors were identified, enabling personalized 5-year risk stratification. The developed model demonstrated robust predictive performance and clinical utility, offering a valuable tool for the individualized assessment and precision management of the 5-year risk of post-operative CRC metastasis. Nevertheless, further validation of its generalizability is warranted through large-scale, prospective studies.

## Data Availability

The raw data supporting the conclusions of this article will be made available by the authors, without undue reservation.
